# School-Based Sedentary Behavior, Physical Activity, and Health-Related Outcomes among Hispanic Children in the United States: A Cross-Sectional Study

**DOI:** 10.3390/ijerph17041197

**Published:** 2020-02-13

**Authors:** Xiangli Gu, Tao Zhang, Senlin Chen, M Jean Keller, Xiaoxia Zhang

**Affiliations:** 1 Department of Kinesiology, University of Texas at Arlington, Arlington, TX 76019, USA; Xiangli.Gu@uta.edu (X.G.); Xiaoxia.Zhang@mavs.uta.edu (X.Z.); 2 Department of Kinesiology, Health Promotion and Recreation, University of North Texas, Denton, TX 76262, USA; Jean.Keller@unt.edu; 3 School of Kinesiology, Louisiana State University, Baton Rouge, LA 70803, USA; Senlinchen@lsu.edu

**Keywords:** ethnic minority, health disparities, health-related quality of life, physical fitness

## Abstract

The main purpose of this study was to examine the relationships between school-based sedentary behavior, physical activity, and health-related outcomes, including cardiorespiratory fitness, weight status, and health-related quality of life (HRQOL) among Hispanic children. The participants were 374 children (192 boys, 182 girls; *M*_age_ = 9.64) recruited from four elementary schools from 3rd grade through to 5th grade. Sedentary behavior and physical activity behaviors (light physical activity [LPA] and moderate-to-vigorous physical activity (MVPA)) during school were measured by accelerometers. Cardiorespiratory fitness and weight status were measured using the FITNESSGRAM^®^, while HRQOL was measured using the PedsQL 4.0^TM^ Spanish version, a validated questionnaire. Sedentary behavior was negatively correlated with cardiorespiratory fitness and HRQOL but positively associated with weight status. MVPA was positively correlated with cardiorespiratory fitness and HRQOL, but negatively associated with weight status and sedentary behavior. Multiple regressions demonstrated that sedentary behavior significantly predicted cardiorespiratory fitness and weight status, whereas MVPA significantly predicted HRQOL. With the current public health priority aiming to reduce health disparities in minority populations, the findings of this study provide important insights. Educators, health care providers, or other professionals working with Hispanic children are encouraged to focus on reducing sedentary behavior and promoting physical activity to improve their health-related outcomes.

## 1. Introduction

Hispanic children are one of the fastest-growing ethnic pediatric populations in the United States (US), representing 24.4% of the population under 18 years of age, and this statistic is projected to increase to 33.5% by 2060 [1]. Hispanic children in the US have a higher obesity prevalence rate and exhibit a greater risk to other health issues, such as diabetes and mental disorders, than Caucasian children [2,3]. Regular participation in physical activity with low sedentary behavior are beneficial to long-term physical and mental health [4]. The substantial differences in body mass index (BMI) and the growing disparities across ethnic and racial groups are public health concerns [2]. More research is needed, however, to address physical activity and health promotion in Hispanic children [5]. Empirical evidence is warranted to understand the relationships among sedentary behavior, physical activity, and health-related outcomes in school-based environments among Hispanic children.

Schools are the ideal settings to decrease sedentary behavior and promote physical activity [6,7] as children spend a substantial amount of awake time there five days a week. Mounting evidence has substantiated the health benefits of meeting the daily 60-min moderate-to-vigorous physical activity (MVPA) recommendation among children and adolescents [8,9]. However, more than half of the children fail to meet this recommendation and spend excessive time in sedentary behavior. Additionally, racial and ethnic differences are even more evident [10]. Schools are imperative settings for ethnic minority children from lower socioeconomic households to be physically active, because they often have less physical activity-related equipment outside of school (at home and in the community) and spend a substantial amount of time on media devices for entertainment [10]. 

Unlike physical activity, excessive prolonged sedentary behavior is negatively associated with physical fitness (e.g., cardiorespiratory fitness) and mental health indicators, such as self-esteem and emotional well-being [11], which should be curbed and reduced through purposeful interventions. According to Mansoubi and colleagues, sedentary behavior refers to non-sleeping or reclining body movements that result in energy expenditure below 1.5 metabolic equivalents (METs) [12]. Hispanic children tend to display a significant amount of sedentary behaviors and therefore are more susceptible to greater health risks [13]. Therefore, it has been a public health priority to reduce health disparities across racial/ethnic groups (e.g., between Hispanic and Caucasian children) through behavioral interventions with a focus on sedentary behavior and physical activity. However, little research has been documented to accurately assess Hispanic children’s sedentary behavior and physical activity behaviors (at a variety of intensities). Quantifying sedentary behavior and physical activity among Hispanic children through valid and objective methods may provide important insights for health promotion in this population.

Sedentary behavior and physical activity are significant behavioral correlates of health-related physical fitness and fatness [14]. According to a recent systematic research review [15], very few studies have focused on the combined effects of sedentary behavior and physical activity on health outcomes (i.e., cardiorespiratory fitness, weight status) among children, and the overall quality of the available evidence was low. Recent evidence also suggests that individuals with high physical activity (especially MVPA) tend to have lower levels of adiposity when compared to those with low physical activity, regardless of their levels of sedentary behavior [16]. In addition, based on the recent FITNESSGRAM^®^ surveillance data [17], around 50–60% (60% of boys and 50% of girls) of fourth and fifth grade children in the US achieved the health fitness zone (HFZ), but this rate declined from middle school to high school [18]. Although overall physical activity participation is positively associated with children’s physical fitness, the association between health-related physical fitness and varying patterns and intensities of physical activity is complicated [8,15]. Specifically, MVPA seems to have strong associations with cardiorespiratory fitness, more so than light intensity physical activity (LPA). 

Sedentary behavior and physical activity may also be related to other health indicators, such as health-related quality of life (HRQOL), particularly among racial/ethnic minority children [15]. HRQOL has been widely used as an indicator of individual mental health, including physical, emotional, social, and school functioning [19]. Recently, a burgeoning number of studies have examined how physical activity and sedentary behavior are associated with HRQOL among children and adolescents. It was indicated that regular participation in physical activity is positively associated with children’s overall HRQOL [20,21]. In a longitudinal study, researchers noted that excessive sedentary behavior offset the positive effect of physical activity on HRQOL [22]. 

To date, few studies have focused on accelerometer-measured sedentary behavior and physical activity in relation to HRQOL among children, especially Hispanic children [8]. More research is also needed to identify the sex and grade differences among the study variables among Hispanic children. Therefore, the main purpose of this study was to examine relationships among school-based sedentary behavior, physical activity, and health-related outcomes, including cardiorespiratory fitness, weight status (BMI), and HRQOL among Hispanic children in 3rd grade through to 5th grade. It was hypothesized that school-based sedentary behavior and physical activity would significantly relate to cardiorespiratory fitness, weight status (BMI), and HRQOL in Hispanic children. The second purpose was to examine the sex (boys vs. girls) and grade (3rd, 4th, or 5th grades) differences among the study variables. It was hypothesized that the study variables would show differences by sex and grade in this Hispanic population. 

## 2. Materials and Methods 

### 2.1. Participants

This study recruited 374 Hispanic children as participants ages 8–11 years (192 boys, 182 girls; Mean age = 9.64 years, SD = 1.16) from four elementary schools in the US. All of the participating schools are located in the same independent school district in Texas displaying similar demographic characteristics. Specifically, the four public elementary schools had similar enrollment sizes (500 to 600) and pupil/teacher ratios (12.1 to 13.2) serving children in kindergarten through to the 5th grade. Before the data collection, the research team met with school principals and classroom teachers to introduce the study purposes and study protocols. Then, recruitment flyers and parents’ consent forms were sent out to 3rd, 4th, and 5th graders’ classrooms in each school in order to randomly recruit participants. A week later, the research team visited the classrooms to collect the returned parental consent forms. There were 159 3rd (81 girls and 78 boys), 112 4th (61 girls and 51 boys), and 103 5th (50 girls and 53 boys) grades children recruited in this study. The Hispanic population within the four schools was approximately 45.7% (SD = 5.6%), and more than 78% of children were categorized as economically disadvantaged. The vast majority of the Hispanic children (92.2%) were eligible for the free or reduced lunch program. The University Institutional Review Board (IRB) approved this study prior to data collection (project identification code: 13224-R15), and informed parental consent and child assent forms were subsequently obtained in accordance with the participating school district and the Declaration of Helsinki. 

### 2.2. Procedure and Design

A cross-sectional research design was used to address the research purposes. Data collection took place during Spring 2015 and Spring 2016, and only participants of Hispanic origin were included in this present data analysis. We measured children’s school-based sedentary behavior and physical activity for five consecutive school days using the Actical accelerometer monitors (Koninklijke Philips Electronics N.V., Amsterdam, The Netherlands). The children’s body height and weight were measured using stadiometers and weight scales, from which BMIs were calculated to indicate the weight status. We further measured the children’s cardiorespiratory fitness using the Progressive Aerobic Cardiovascular Endurance Run (PACER) from the FITNESSGRAM^®^ test battery [23]. After that, we administered the Hispanic-version HRQOL standardized questionnaire to the children within the classrooms. At each survey administration, a trained research assistant read each question item to the children in English and then the classroom teachers, who were bi-lingual, repeated the instructions in Spanish. The children were instructed to honestly answer the HRQOL questions and they were assured that their responses would be kept anonymous and not affecting their grades. The children spent approximately 10–15 min completing the HRQOL questionnaire.

### 2.3. Measurements

#### 2.3.1. Socio-Demographic Variables

Data for age, sex, and race/ethnicity were obtained from the school district. Socioeconomic status (SES) was identified based on each participant’s lunch meal status according to the Income Eligibility Guidelines, which were also shared by the participating schools.

#### 2.3.2. Sedentary Behavior and Physical Activity

Actical activity monitors were used to objectively measure the children’s school-based sedentary behavior, LPA, and MVPA for five consecutive school days. Trained research assistants equipped each participant with an Actical accelerometer on their non-dominant wrist. All accelerometers were marked with an identification number and the time of wearing was recorded. Accelerometers were set up using 60 s epochs after entering personal information, including age, sex, height, and weight. Standard Activity Energy Expenditure (AEE) cut-points for sedentary behavior (AEE <  0.01  kcal/kg/min), LPA (0.01  ≤  AEE  <  0.04  kcal/kg/min), and MVPA (AEE ≥ 0.04  kcal/kg/min) were used to determine the time spent in each activity category [24,25]. The average minutes spent in sedentary behavior, LPA, and MVPA were calculated by dividing the total number of minutes accumulated during the school hours (~7 h) over the five days by the days accelerometers were worn.

#### 2.3.3. Weight Status and Cardiorespiratory Fitness

The children’s height and weight were measured using stadiometers and weight scales, and BMI was calculated by dividing weight (in kilogram) by squared height (in meter). The Progressive Aerobic Cardiovascular Endurance Run (PACER) was used to measure cardiorespiratory fitness using the FITNESSGRAM^®^ test battery [23]. The PACER laps were converted into VO2max as the index of cardiorespiratory fitness in this study. Specifically, VO2max was determined based on the total number of successful PACER “laps” children completed after taking into account their age, sex, and BMI based on the predictive equation: VO2max = 41.77 ± (PACERlaps* 0.49) − 0.0029 *(PACERlaps^2) − 0.62 * BMI ± 0.35 *Age * Sex [26].

#### 2.3.4. Health-Related Quality of Life (HRQOL)

The PedsQL 4.0^TM^ Spanish version [19] was used to assess children’s HRQOL, including physical functioning (8 items), emotional functioning (5 items), social functioning (5 items), and school functioning (5 items). The PedsQL items use a 5-point Likert-type scale to assess the frequency of functioning problems (0 = never a problem; 1 = almost never a problem; 2 = sometimes a problem; 3 = often a problem; 4 = almost always a problem). The items were reverse coded and then transformed to a linear scale (0–100). The total composite score was used in this study, with 100 indicating the highest and 0 indicating the lowest possible HRQOL. This instrument has previously demonstrated sufficient reliability and validity in assessing children populations [9,19] and acceptable internal consistency in this present study (Cronbach’s alpha = 0.77).

### 2.4. Data Analysis

After screening the raw data for accuracy and normality, the expectation-maximization (EM) approach was used to treat missing values and Little’s MCAR (Missing Completely At Random) test was used to test if data were completely missing at random. Although the EM data imputation showed generally unbiased correlation and regression coefficients, a conservative critical alpha value was used in interpreting the results (e.g., 0.01 instead of 0.05). Subsequently, three steps were taken using SPSS 25.0 (SPSS Inc., Chicago, IL, USA). First, descriptive statistics (mean, standard deviation) and a 2 × 3 multivariate analysis of variance (MANOVA) was conducted to test the sex (boys vs. girls) and grade (3rd, 4th, or 5th grades) differences in the study variables. Secondly, Pearson product-moment correlation analyses were conducted to examine the associations among the study variables (i.e., sedentary behavior, LPA, MVPA, cardiorespiratory fitness, BMI, and HRQOL). Finally, three hierarchical multiple regressions were conducted to determine the roles of sedentary behavior (Step 3) and physical activity (Step 2: LPA and MVPA) on the three health-related outcomes, including cardiorespiratory fitness, BMI, and HRQOL, after accounting for the demographic variables (step 1: sex and grade).

## 3. Results

Table 1 shows the descriptive results for the study variables by sex (boys vs. girls) and grade (3rd, 4th, and 5th). The majority of the Hispanic children in this study met the 60 min of MVPA recommendation per day during school hours, while 7.5% of 3rd grade, 5.4% of 4th grade, and 8.7% of 5th grade children failed to reach the national daily MVPA recommendation. The MANOVA results yielded a significant main effect for sex (boys and girls; F6, 363 = 20.09, *p* < 0.01, $\eta_{p}^{2}$ = 0.25) and grade (3rd, 4th and 5th grade; F12, 726 = 26.70, *p* < 0.01, $\eta_{p}^{2}$ = 0.31), but no sex and grade interaction (*p* > 0.05). Boys demonstrated higher school-based MVPA (F1, 374 = 15.06; *p* < 0.01, $\eta_{p}^{2}$ = 0.04] and cardiorespiratory fitness (F1, 374 = 17.95; *p* < 0.01, $\eta_{p}^{2}$ = 0.05), but girls engaged in more LPA (F1, 374 = 28.48; *p* < 0.001, $\eta_{p}^{2}$ = 0.07). By grade, 5th grade children showed significantly higher BMI than the 3rd and 4th grade children (F2, 374 = 5.19; *p* < 0.01, $\eta_{p}^{2}$ = 0.03), but no significant grade difference was observed for cardiorespiratory fitness. In addition, 4th and 5th grade children reported higher HRQOL scores than 3rd grade children (F2, 374 = 8.38; *p* < 0.001, $\eta_{p}^{2}$ = 0.04). They also showed a higher MVPA time, higher sedentary behavior (F2, 374 = 55.03; *p* < 0.001, $\eta_{p}^{2}$ = 0.23), but lower LPA compared with 3rd graders (F2, 374 = 10.41; *p* < 0.01, $\eta_{p}^{2}$ = 0.05).

Table 2 shows the results from the Pearson product–moment correlation analysis. The results demonstrated that sedentary behavior was negatively correlated with cardiorespiratory fitness (r = −0.16, *p* < 0.01) and HRQOL (r = −0.18, *p* < 0.01) but was positively associated with BMI (r = 0.22, *p* < 0.01). There were small but significant correlations between MVPA and cardiorespiratory fitness (r = 0.15, *p* < 0.01) and HRQOL (r = 0.26, *p* < 0.01), respectively. The strong but negative correlation of MVPA with sedentary behavior and LPA (r = −0.42 and −0.67, respectively) were also observed in this study. It was found that BMI had a small but significant relationship with MVPA (r = −0.11, *p* < 0.05), but not with LPA in this study.

Our data screening process based on the expectation-maximization (EM) approach indicated that the data were completely missing at random (Little’s MCAR test χ^2^ (27) = 21.14, *p* = 0.78). The assumption of non-multicollinearity was met for all study variables (VIFs = variance inflation factor) were close to 1 and Tolerance statistics were greater than 0.20). Subsequently, three hierarchical multiple regression analyses were conducted, and the results are presented in Table 3. After adjusting for sex and grade in the models, sedentary behavior was a significant negative predictor of cardiorespiratory fitness and BMI regardless of the physical activity levels, accounting for 7.6% and 6.4% of the variances, respectively. MVPA, but not sedentary behavior, significantly predicted HRQOL after controlling for sex and grade.

## 4. Discussion

The major purpose of this study was to examine the relationships among school-based accelerometer-measured sedentary behavior, different intensity levels of physical activity, and health-related outcomes among Hispanic children. Our findings demonstrate that sedentary behavior was a negative correlator, but MVPA was a positive correlator of the three health-related outcomes (i.e., cardiorespiratory fitness, weight status, and HRQOL) among Hispanic children. This study examined the potential offsetting effect of physical activity and sedentary behavior on health-related outcomes by controlling for potential covariates (sex and grade). With current public health priority aimed to reduce health disparities across ethnic groups, the findings of this study provided important insights for health promotion among Hispanic boys and girls during elementary school years.

The MANOVA results revealed significant sex and grade effects on some of the health-related outcomes. Specifically, significant sex effect for cardiorespiratory fitness and physical activity was observed in this Hispanic children sample, confirming the prior finding that boys generally are more active and physically fit than girls [9]. Similarly, we also observed a significant grade difference for sedentary behavior (increasing trend) and physical activity (decline trend), which is consistent with the age- or grade-based disparity observed in a prior study [11]. Although most of the Hispanic children in this study were physically active during school hours, sedentary behavior increased in 4th (12.5%) and 5th (26.2%) graders. Concomitant with the increasing trend in sedentary behavior, we also observed a significant linear trend for BMI with 50%, 42%, and 55.3% of the children being classified as overweight/obese (85th percentile) in 3rd, 4th, and 5th grades, respectively. This trend is also shown in the NHANES 2007-2008 data [27]. HRQOL also showed a significant difference by grade, which was lower among younger children (i.e., 3rd graders) than in older children (i.e., 4th or 5th graders). The lower HRQOL in younger Hispanic children might have reflected the compromised psychological and physical health [28,29], suggesting the need for health and educational interventions during early childhood.

This study provides evidence that decreasing sitting time and increasing MVPA time have the potential to improve physical and mental health among Hispanic children. Specifically, sedentary behavior was found to be negatively associated with cardiorespiratory fitness but positively related to body weight, regardless of the intensity levels of physical activity. This finding is consistent with a recent study made on United Kingdom children of ages 10–11, which suggested that school day activities, including MVPA and sedentary behavior, significantly predicted adiposity and cardiorespiratory fitness, but not HRQOL [16]. Our results further found that MVPA, not LPA or sedentary behavior, was a significant correlate of HRQOL among Hispanic children, supporting existing evidence that achieving the recommended 60-min MVPA per day is more likely associated with better HRQOL among children [9,20]. This finding is in line with Dumuid and colleagues’ observation in their 12-nation cross-sectional observational study that HRQOL was significantly related to MVPA (relative to remaining behaviors: sedentary behavior and sleep) [20]. Recent reviews also suggest that optimal health improvements are likely to be seen when sedentary behavior is replaced by MVPA [15]. The above findings suggest that additional future studies are needed to simultaneously examine sedentary behavior and physical activity to thoroughly understand the behavioral mechanism of health-related outcomes among Hispanic children.

One major strength of this study is the adoption of objective measures to assess school-based sedentary behavior, different intensity levels of physical activity, cardiorespiratory fitness, and weight status (BMI). This study involved a large Hispanic children sample recruited from four elementary schools to inform the behavioral mechanism of health-related outcomes, which was represented by both physical (i.e., cardiorespiratory fitness and weight status [BMI]) and mental health indicators (i.e., HRQOL). However, there are several limitations. First, despite the large sample size, the findings may only be generalizable to Hispanic children and schools of similar characteristics. Second, although the Actical accelerometer objectively measured school-based sedentary behavior in this study, a more comprehensive assessment of sedentary behavior may be needed to capture various types of sedentary behaviors (i.e., screen time, computer use, sitting, etc.). Finally, this study utilized the cross-sectional research design; therefore, causal relationships among the study variables cannot be claimed. Future research should employ longitudinal or experimental research designs.

## 5. Conclusions

In summary, this study is one of the first studies to examine the relationships and differences among school-based sedentary behavior, physical activity, and health-related outcomes in Hispanic children. Findings from the current study indicate that health promotion through supporting physical activity and decreasing sedentary behavior in this population is important and needed during school hours. School educators, health care providers, or other professionals working with Hispanic children are encouraged to focus on reducing sedentary behavior and promoting physical activity to improve health-related outcomes, including cardiorespiratory fitness, weight status, and HRQOL among Hispanic children. Culture- and sex/gender-tailored strategies may be needed to maximize the effectiveness of these efforts.

## Figures and Tables

**Table 1:** Descriptive results of the study variables by sex and grade (N = 374).

Variables	Boys N = 192	Girls N = 182	3rd Grade N = 159	4th Grade N = 112	5th Grade N = 103
	M(SD)	M(SD)	M(SD)	M(SD)	M(SD)
Sedentary behavior (minutes)	41.54 (1.75)	38.03 (1.80)	23.13 (1.89)	42.63 (2.26)	53.60 (2.35)
LPA Time (minutes)	258.75 (2.14)	275.10 (2.19)	276.02 (2.30)	264.31 (2.76)	260.45 (2.86)
MVPA Time (minutes)	108.69 (2.36)	95.56 (2.42)	95.95 (2.55)	106.36 (3.04)	104.05 (3.16)
Cardiorespiratory fitness	39.86 (0.38)	37.57 (0.39)	39.01 (0.41)	38.80 (0.49)	38.34 (0.51)
BMI (kilograms/meters^2^)	21.68 (0.36)	20.74 (0.37)	20.50 (0.39)	20.71 (0.47)	22.42 (0.49)
HRQOL	76.65 (0.99)	74.66 (1.02)	72.97 (1.07)	79.65 (1.28)	74.34 (1.33)

Note. LPA = light physical activity; MVPA = moderate-to-vigorous physical activity; BMI = body mass index; HRQOL = health-related quality of life; M (SD) = mean (standard deviation). Within-subjects contrast for sex: Wilks’ Lambda F6, 363 = 20.09, *p* < 0.01, η^2^ = 0.25; within-subjects contrast for grade: Wilks’ Lambda F12, 726 = 26.70, *p* < 0.001, η^2^ = 0.31; no sex by grade interaction: Wilks’ Lambda F12, 726 = 0.71, *p* = 0.75, η^2^ = 0.01.

**Table 2:** Pearson product–moment Correlation among the study variables (N = 374).

Variables	1	2	3	4	5	6
1. Sedentary behavior (minutes)	-					
2. LPA Time (minutes)	−0.20 **	-				
3. MVPA Time (minutes)	−0.42 **	−0.67 **	-			
4. Cardiorespiratory fitness	−0.16 **	−0.07	0.15 **	-		
5. BMI (kilograms/meters^2^)	0.22 **	−0.01	−0.11 *	−0.73 **	-	
6. HRQOL	−0.18 **	−0.08	0.26 **	0.18 **	−0.16 **	-

Note. LPA = light physical activity; MVPA = moderate-to-vigorous physical activity; BMI = body mass index; HRQOL = health-related quality of life. * *p* < 0.05; ** *p* < 0.01.

**Table 3:** Results of three hierarchical regressions for the health-related outcomes (N = 374).

Dependent Variable	Step #	Independent Variable	R^2^	B	β	t
Cardiorespiratory fitness	Step 1		0.047 **			
	Step 2		0.064 *			
	Step 3		0.076 *			
		Sex		−2.22	−0.21 **	−4.02
		Grade		0.25	0.04	0.59
		LPA		−0.01	−0.08	−0.76
		MVPA		−0.01	−0.04	−0.36
		Sedentary Behavior		−0.04	−0.22 **	−2.12
BMI	Step 1		0.031 **			
	Step 2		0.054 *			
	Step 3		0.064 *			
		Sex		−1.02	−0.10	−1.92
		Grade		0.43	0.07	1.04
		LPA		0.02	0.09	0.88
		MVPA		0.00	0.02	0.14
		Sedentary Behavior		0.04	0.21 *	1.99
HRQOL	Step 1		0.01			
	Step 2		0.094 **			
	Step 3		0.094			
		Sex		−1.65	−0.06	−4.02
		Grade		1.29	0.08	0.59
		LPA		0.09	0.19	−0.76
		MVPA		0.15	0.36 **	−0.36
		Sedentary Behavior		−0.01	−0.03	−2.12

Note. LPA = light physical activity; MVPA = moderate-to-vigorous physical activity; BMI = body mass index; HRQOL = health-related quality of life. F-test and R^2^ were conducted to assess model fit. R^2^ values are cumulative, with each incremental step adding to the variance explained; β values are standardized regression coefficients at each step of the regression analysis. * *p* < 0.05; ** *p* < 0.01.
